# Copresence of High-Risk Human Papillomaviruses and Epstein–Barr Virus in Colorectal Cancer: A Tissue Microarray and Molecular Study from Lebanon

**DOI:** 10.3390/ijms22158118

**Published:** 2021-07-29

**Authors:** Karim Nagi, Ishita Gupta, Nawaf Jurdi, Amber Yasmeen, Semir Vranic, Gerald Batist, Ala-Eddin Al Moustafa

**Affiliations:** 1 College of Medicine, QU Health, Qatar University, Doha 2713, Qatar; ishita.gupta@qu.edu.qa (I.G.); svranic@qu.edu.qa (S.V.); 2 Biomedical and Pharmaceutical Research Unit, QU Health, Qatar University, Doha 2713, Qatar; 3 Department of Pathology and Laboratory Medicine, American University of Beirut Medical Center, Beirut 1107, Lebanon; nj14@aub.edu.lb; 4 Segal Cancer Center/Lady Davis Institute for Medical Research, JGH/McGill University, Montreal, QC H3A 0G4, Canada; amber.yasmeen@mail.mcgill.ca (A.Y.); gerald.batist@mcgill.ca (G.B.); 5 Oncology Department, Faculty of Medicine, McGill University, Montreal, QC H3A 0G4, Canada; 6 Biomedical Research Centre, QU Health, Qatar University, Doha 2713, Qatar

**Keywords:** Epstein–Barr virus, human papillomavirus, colorectal cancer, tumor grade, Lebanon

## Abstract

Colorectal cancer (CRC) is the third most common cause of cancer-related deaths worldwide. Human papillomaviruses (HPVs) and Epstein–Barr virus (EBV) have been reported to be present in different types of human cancers, including CRCs, where they can play a key role in the onset and/or progression of these cancers. Thus, we herein explored the prevalence of high-risk HPVs and EBV in a cohort of 94 CRC tissue samples and 13 colorectal normal tissues from the Lebanese population using polymerase chain reaction, immunohistochemistry, and tissue microarray methodologies. We found that high-risk HPVs are present in 64%, while EBV is present in 29% of our CRC samples. Additionally, our data showed that high-risk HPV types (16, 18, 35, 58, 51, 45, 52, 31, and 33) are the most frequent in CRC in the Lebanese cohort, respectively. Our data point out that HPVs and EBV are copresent in 28% of the samples. Thus, this study clearly suggests that high-risk HPVs and EBV are present/copresent in CRCs, where they could play an important role in colorectal carcinogenesis. Nevertheless, further investigations using a larger cohort are needed to elucidate the possible cooperation between these oncoviruses in the development of CRC.

## 1. Introduction

Colorectal cancer (CRC) is the third most commonly diagnosed form of cancer worldwide, with more than 1.9 million new cases in 2020, which accounts for approximately 10% of all cancer cases according to the World Health Organization GLOBOCAN database [1]. CRC is considered the fourth primary cause of cancer-related deaths in males and the third foremost cause of cancer-related mortality among females [1]. CRCs are classified as inherited, inflammatory, or sporadic [2]. Sporadic cancers account for around 80% of all CRCs, and current investigations aim to identify the underlying causative factors for the onset of sporadic CRCs [2]. Along with environmental (diet, obesity) [3,4] and genetic factors, several infectious agents, including human papillomaviruses (HPVs), are considered risk factors [5].

Around 20% of all cancers are known to be caused by infectious agents including bacterial and viral components [5]. Viral infections by small DNA viruses including polyomaviruses, HPVs, and Epstein–Barr virus (EBV) are known to be involved in several carcinomas such as Merkel cells, cervical cancer, Burkitt’s lymphoma, Hodgkin’s lymphoma, and nasopharyngeal carcinomas [4,5,6,7,8,9].

HPVs are nonenveloped, double-stranded DNA viruses that infect the basal layer of epithelial cells [5,10]. HPVs are divided into two types: low-risk and high-risk HPVs [11]. High-risk HPVs are associated with the onset and development of various epithelial cancers including cervical, anal, penile, esophageal, and vaginal [12,13,14,15,16]; however, the carcinogenic probability of each strain differs. Of all HPVs, types 16 and 18 are well-established carcinogenic viruses [17]. HPVs consist of early (E1, E2, E4, E5, E6, and E7) and late proteins (L1 and L2) [18]. The early protein E5 binds and activates epidermal growth factor receptor-1 (EGFR-1), while E6 and E7 act by impeding the activity of tumor suppressor genes p53 and pRb, respectively, through their targeted destruction by various pathways, which could lead to precancer [19,20]. High-risk oncoproteins of HPVs E5 and E6/E7 can cooperate to initiate epithelial-to-mesenchymal transition (EMT), which is a hallmark of cancer progression [21]. Moreover, it has been demonstrated that E6/E7 oncoproteins of high-risk HPVs convert noninvasive and nonmetastatic cancer cells into invasive and metastatic ones [22]. Several studies revealed that high-risk HPVs are present in human CRCs worldwide including few countries of the Middle East (ME) region [23,24,25,26].

On the other hand, Epstein–Barr virus (EBV), entails six EBV nuclear proteins (EBNA1, -2, -3A, -3B, -3C, and -LP), three latent membrane proteins (LMP1, -2A, and -2B), and multiple noncoding RNAs (EBERs and miRNAs) [27,28,29]. EBV-induced cancers involve EBV’s latent cycle; these latent EBV forms (e.g., EBNA-1, EBERs, LMP-1) regulate the four types of latent gene expression (latency I, II, III, and IV) [30]. Three latency programs (Latency I, II, and III) exemplify the types of cancers associated with EBV [30,31]; latency I is frequently found in Burkitt lymphoma, while latency II is associated with nasopharyngeal carcinoma, Hodgkin’s disease, and T-cell non-Hodgkin’s lymphomas, while latency III generally affects immunocompromised patients [30]. Suppressed state of EBV can develop to cause several B-cell lymphomas, oral carcinomas (especially nasopharyngeal), gastric cancer, and other malignancies [30,31]. It is known that LMP1, an oncogenic protein of EBV, enhances cellular growth, inhibits apoptosis, and promotes cell motility and angiogenesis in different types of human carcinomas [32,33]. To date, there are few studies documenting the presence and/or role of EBV in human CRCs [34,35,36,37,38,39,40,41].

Recent investigations, including ours, pointed out that oncoviruses (high-risk HPVs and EBV) can be copresent in several types of human cancers, including CRCs, where they can cooperate in the initiation and progression of these cancers via the crosstalk of their oncoproteins [42,43,44,45,46]. Recently, copresence of HPVs and EBV was reported in 16% of CRCs samples from Bosnia and Herzegovina [47]; however, the copresence of HPVs and EBV in CRCs in the ME region is limited to only one study from Syria [46]. This study revealed that 17% of the 102 cancer samples were positive for both of these oncoviruses [46]. Thus, we herein explore the copresences of high-risk HPVs and EBV in CRC samples from Lebanon. Our study points out that HPVs and EBV are copresent in 28% of the samples.

## 2. Results

### 2.1. Clinicopathological Characteristics of the Cohort

The study included 107 CRC samples from 37 (35%) male and 70 (65%) female patients. The mean age of patients is 60 years (range, 3–89 years). The plurality of specimens was taken from the rectosigmoid colon (46 cases, 49%), while the remaining samples originated from other anatomic parts of the colon (48 cases, 51%). Table 1 summarizes the clinicopathological characteristics of the cohort.

All tumors in the present study were histologically confirmed as adenocarcinomas. Of the studied cases, the majority of cancers (72 cases, 77%) were grade 1 (low-grade), while 22 cases (23%) were grade 2 (high-grade) (Table 1). Based on the pT stage, 6 (6.4%) cases were stage 1, 5 (5.3%) cases stage 2, 79 (84%) cases stage 3, and 4 (4.3%) cases were reported to have pT4 stage disease at the time of presentation. Lymph node metastases were found in 42 cases (44.7%) (Table 1).

### 2.2. Presence of High-Risk HPV Subtypes and EBV by PCR

**Figure 1:**
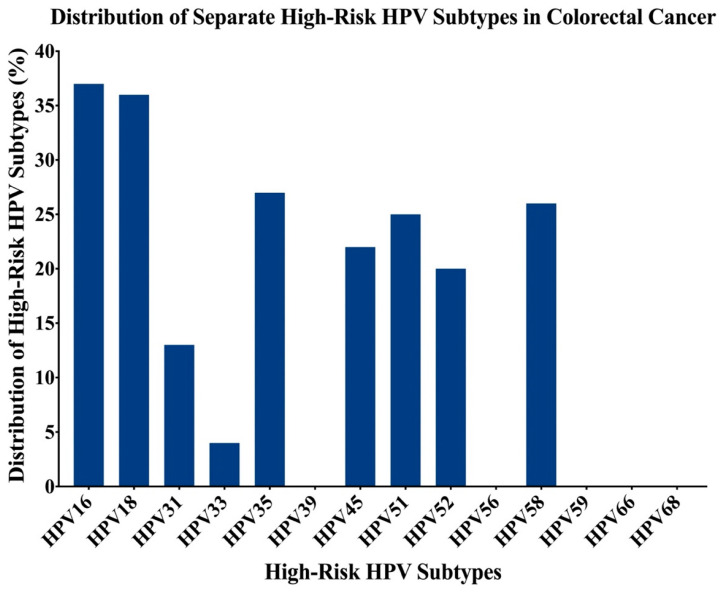
The distribution of each high-risk HPV subtype in Lebanese colorectal cancer samples. The PCR analysis included 94 colorectal cancer (CRC) samples revealing that the most frequent human papillomaviruse (HPV) subtypes are 16, 18, 35, 58, 51, 45, 52, 31, and 33.

In total, 107 (13 normal and 94 cancer) colorectal samples had interpretable results for HPV and EBV by PCR. We found that 60 of the 94 CRC samples were positive for high-risk HPVs (63.8%); the most commonly identified high-risk HPVs in the cohort (*n* = 94) was HPV16 (39%), followed by HPV18 (38%), HPV35 (29%), HPV58 (28%), HPV51 (26%), HPV45 (23%), HPV52 (21%), HPV31 (14%) and HPV33 (4%) (Figure 1). HPV types 39, 56, 59, 66, and 68 were not detected in our examined samples (Figure 1).

On the other hand, 4 of the 13 CR normal tissues (31%) were positive for HPV; however, a significant difference was noted in the incidence of HPV infection between CRC cases and controls (*p* = 0.03). Of the 4 CR normal samples, none of them expressed more than one HPV type.

Furthermore, our data pointed out that 9 (9.6%) CRC cases were positive for two HPV subtypes. Three or more coinfections were also detected in 40/94 (42.5%) CRC samples; 8 cases (8.5%) had 3 HPV coinfections, 11 cases (11.7%) had 4 coinfections, 13 cases (13.8%) had 5 coinfections, and 8 cases (8.5%) revealed the presence of 6 high-risk HPV coinfections. The most commonly identified combination was HPV16/18, where 19 (20.2%) of the 94 CRC samples were coinfected with both HPV16 and 18.

Regarding the presence of EBV, we noted that 27/94 (28.7%) of the CRC samples were positive for this oncovirus, while all normal colorectal samples were negative for EBV (*p* = 0.03). Interestingly, our data revealed that while all normal colorectal samples were negative for the copresence of HPV and EBV, 26 (27.6%) cancer samples were positive for both high-risk HPVs and EBV (*p* = 0.03). However, we noted a significant association between the presence of EBV and HPV types-18 (*p* < 0.0001), -33 (*p* = 0.006), -35, -52 and -58 (*p* < 0.0001, each) in our cohort of CRC samples (*χ*^2^ test) (Table 2).

Based on the anatomical regions, 33/46 (71.7%) rectosigmoid cancer cases were positive for HPVs, while EBV was present in 12/46 (26.1%) of these cases. Moreover, 12/46 (26.1%) rectosigmoid cases were positive for both high-risk HPVs and EBV. On the other hand, 27/48 (56.3%) of the other parts of the colon cancer cases were positive for HPVs, and 15/48 (31.3%) were positive for EBV. In addition, we found that 14/48 (29.2%) of the other parts of the colon cancer cases exhibit copresence of HPV and EBV. However, we found no significant difference between the presence of HPV or/and EBV and anatomical locations.

### 2.3. Expression Patterns of E6 and LMP1 Oncoproteins of High-Risk HPVs and EBV

**Figure 2:**
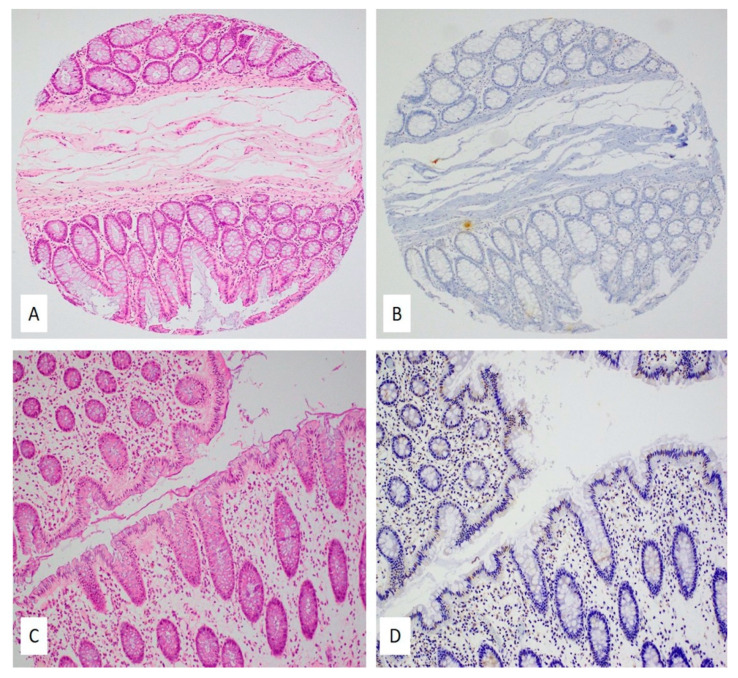
(**A**–**D**) HPV and EBV expression in normal colonic mucosa; (**A**,**C**) hematoxylin and eosin slides (H&E) of two cases with normal colonic mucosa, 10×; (**B**,**D**) corresponding IHC slides for E6 protein (**B**) and LMP1 protein (**D**) revealed no protein expression in the normal colonic epithelium; however, some intramucosal lymphocytes were positive for LMP1 protein (10×). Immunohistochemical staining was performed using the standardized avidin–biotin immunoperoxidase method.

**Figure 3:**
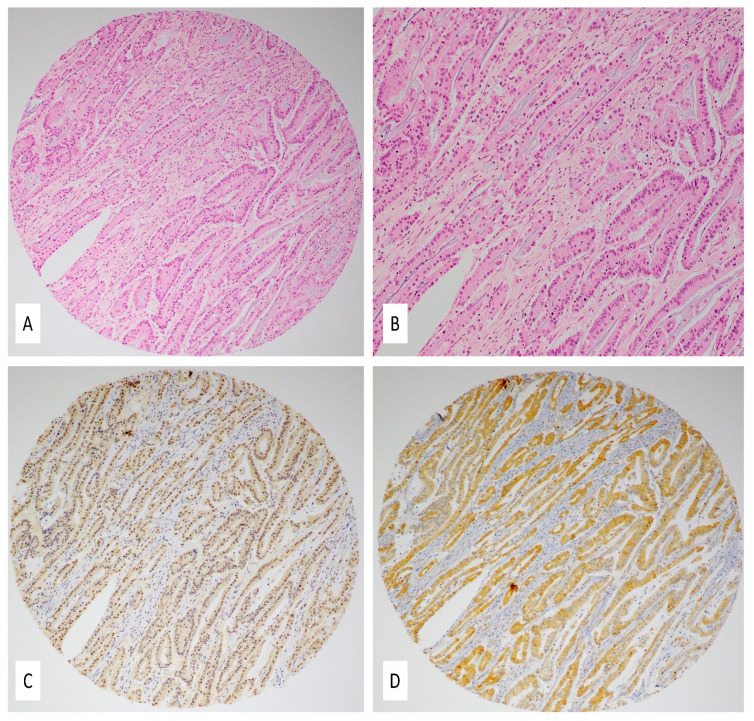
(**A**–**D**) Coexpression of EBV and HPV in colorectal carcinoma: (**A**,**B**) hematoxylin and eosin slides (H&E) showing a moderately differentiated colorectal carcinoma (magnifications 10× and 20×, respectively); (**C**,**D**) IHC revealed diffused and strong LMP1 (**C**) and E6 protein expression (**D**) (10×). Immunohistochemical staining was performed using the standardized avidin–biotin immunoperoxidase method.

Figure 2 and Figure 3 are representative images showing IHC expression patterns of E6 and LMP1 proteins in normal colonic mucosa (Figure 2) and colorectal carcinomas (Figure 3). Figure 2A,C (normal mucosa) and Figure 3A,B (colorectal carcinoma) are the corresponding hematoxylin and eosin (H&E) images.

Among the 94 CRC samples used for IHC to detect E6 (high-risk HPV) and LMP1 (EBV), 22 of the tissue microarray (TMA) blocks lacked cancerous tissues, plausibly due to the lack of exact H&E/FFPE matching required for normal TMA construction. Moreover, 45 of the remaining 72 CRC cases (62.5%) were positive for E6 of high-risk HPVs, as revealed by IHC analysis above the threshold of 1% of positive cancer cells (Figure 3D). On the other hand, LMP1 of EBV was expressed in 20/72 (27.7%) of the CRC cases (Figure 3C). Only one normal mucosa was positive for HPV, while three normal cases had epithelium positive for EBV; intramucosal lymphocytes were also occasionally positive for LMP1 protein (Figure 2B,D); however, although there was a significant difference in the incidence of HPV infection between CRC cases and controls (*p* = 0.0008), whereas there was no significant difference in EBV infection between CRC cases and controls (*p* = 0.99). Copresence of HPV (E6) and EBV (LMP1) was detected in 14/72 (19.4%) CRC cases; similarly, in our PCR data, we noted the copresence of both oncoviruses in 27/94 (28.7%) of the CRC cases.

Finally, we should highlight that IHC and PCR data for HPV and EBV are in good correlation (62.5 vs. 63.8% for HPV and 27.7% vs. 28.7% for EBV, respectively). Nevertheless, there is a slight discrepancy in the detection of the copresence of HPV and EBV by IHC and PCR. Such discrepancy is shared by previous studies in their results pertaining to PCR and IHC while reporting HPV and EBV positivity in cancer cases [50,51,52]. Hence, the discrepancy can be attributed to variation in sensitivity and reliability between PCR and IHC in detecting HPV and EBV among patient samples.

### 2.4. Correlation of Clinicopathological Characteristics with HPV/EBV Positivity

In our cohort, we found no correlation between HPV positivity and tumor grade (*p* = 0.78), stage (*p* = 0.48), lymph node involvement (*p* = 0.63), or anatomical location (*p* = 0.18). Moreover, there was no correlation between EBV positivity and tumor grade (*p* = 0.65), stage (*p* = 0.96), lymph node involvement (*p* = 0.15), or anatomical location (*p* = 0.74).

The copresence of high-risk HPVs and EBV (HPV+/EBV+) did not correlate with tumor grade (*p* = 0.75), stage (*p* = 0.96), lymph node involvement (*p* = 0.21), or anatomical location (*p* = 0.81). This may be attributed to the small number of samples in the study.

## 3. Discussion

To the best of our knowledge, this is the first study reporting the presence/copresence of high-risk HPVs and EBV in human CRC in the Lebanese population. Our data revealed that high-risk HPVs are present in 64% of CRC samples, while normal colorectal samples revealed positivity for high-risk HPVs in 4 of 13 cases; however, a significant difference in HPV-positivity was detected between normal and tumor colorectal tissue samples (*p* = 0.03). In concordance with data obtained in the present study, a large number of investigations worldwide revealed that high-risk HPVs are present in around 40–80% of human CRC cases [53,54,55,56]. While in the ME region, the presence of HPVs is limited to few studies; for instance, a report from Turkey showed a high HPVs prevalence (81%) in CRC [26]. In addition, other countries from the ME region such as Syria and Iran reported a comparatively lower frequency of HPV prevalence (33–37%) [46,57,58,59,60,61]. However, an investigation conducted in Saudi Arabia reported a very low prevalence of HPV (~1%) in CRC samples [62]. On the other hand, and regarding HPV types in human cancers in Lebanon, previous reports revealed that the presence of HPVs varies from 6% to 76% in oropharyngeal and cervical cancers with HPV16 being the most prominent [63,64]. In our study, we found that the most frequent HPV types in CRC samples are HPVs-16, -18, -35, -58, -51, -45, -52, -31, and -33, in descending order. Similar to our data, studies from other countries in the ME region, reported HPVs-16, -18, -51, and -58 as the most prevalent high-risk HPV subtypes in CRC [59,60,65,66]. More specifically, our data are similar to data obtained in CRCs from Iran, Israel, Syria, and Turkey, where HPVs-16, -18, -31, -33, and -35 were reported as the most prominent high-risk HPV subtypes [23,46,59,60,61,67]. Furthermore, to understand the role of HPV in CRC pathogenesis, a study by Motlagh et al. [65] and Chen et al. [57] indicated that E6 oncoprotein of HPV type 16 silences p53 expression. Their analysis reported loss of p21 and mdm2 mRNA expression levels in E6-positive/p53-mutated tumors, in comparison with adjacent normal tissues [57]. The study reports that the presence of E6 oncoprotein inactivates p53, thus reducing the expression levels of p21 and mdm2 [57], indicating a plausible underlying mechanism of the role of HPV in CRC pathogenesis. Moreover, a study from our group revealed that E6/E7 of HPV type 16 can transform human normal mesenchymal CR cells; thus, E6/E7 enhances the cell migration ability of these transformed cells [68]. Another investigation from our lab pointed out that E6/E7 of HPV type 16 can convert noninvasive and nonmetastatic cancer cells into invasive and metastatic ones [22]. Thus, it is likely that high-risk HPVs play an important role in the development and/or progression of human carcinomas including colorectal. On the other hand, our data showed HPV infection to be prevalent in the rectosigmoid junction and colon; this observation is consistent with the transmission of HPV to different anatomical locations through peripheral blood lymphocytes [57]. However, in concordance with other studies, we did not find any correlation between HPV infection and the anatomic location of tumors [57,69].

Vis à vis EBV in CRC, a few studies have reported EBV positivity in CRC cases ranging from 20–50% [35,39,40,70,71,72,73,74,75]; however, other studies failed to detect the presence of EBV in human CRC samples [41,70,72,76,77,78]. Accordingly, our present study in the Lebanese population showed that 27 (29%) of the 94 samples are EBV positive. We also found sporadic EBV positivity in adjacent normal mucosa as well as in intramucosal lymphocytes. Our results are in accordance with data obtained using PCR and IHC in CRC samples from Bosnia (25%) [47], China (28%) [39], South Korea (31%) [78], and Syria (36%) [46]. However, while few studies reported a low prevalence of EBV positivity (1–8%) [79,80,81], other studies such as those in New Guinean [82] and Italy [73] reported a prevalence of ~40–50%. In addition, it should be noted that other investigations from the Middle East [72] and other parts of the world [41,70,76,77,78,83,84] failed to detect EBV in adenocarcinoma of the colon.

In our present study, we explored the copresence of high-risk HPVs and EBV in human CRC cases in the population of Lebanon. Interestingly, it has been recently pointed out that these oncoviruses can be copresent in several types of human cancers including cervical, oral, breast, and CRC, and their copresence varies from 12 to 50% of the cases [42,43,44,45,46,47,75,85,86,87,88,89,90,91]. Although, various studies reported an association between EBV presence and CRC [35], evidence on the role of EBV in CRC is contradictory since the majority of studies that indicate a positive association are based on PCR or IHC assays, alone or in combination with other techniques. Nevertheless, studies based on PCR assay can plausibly be contaminated by EBV-positive inflammatory cells (lymphocytes), suggesting the presence of EBV in its latent form (LMP1) and not lytic form in lymphoid aggregates within the tumor mass [70]. However, both techniques may be biased as they primarily select/target a certain EBV gene (different primers) or protein (different antibodies); therefore, other methods such as NGS assays or in situ hybridization of EBER that have higher sensitivity should be used to detect EBV in colorectal and other cancers [92]. Given the presence of common nuclear expression of LMP1 protein in our cohort (in both cancer cells and lymphocytes), future work should definitely include positive immunostains (controls) such as nasopharyngeal carcinoma and classical Hodgkin lymphoma tissues. In addition, a confirmatory in situ hybridization assay for EBER should further validate our results (this assay is currently unavailable in our lab due to technical difficulties).

In addition, it has been demonstrated that the copresence of HPVs and EBV is associated with tumor grade and stage in human carcinomas [43,45,46,47,91]. Accordingly, we found in our study that HPVs and EBV are copresent in 28% of the CRC samples in Lebanon. However, there was no significant association between HPVs and EBV copresence with tumor grade or stage, which could be due to the limited number of samples used herein. On the other hand, it is postulated that EBV infection can cooperate with other oncoviruses in the development and progression of several human carcinomas [34,93,94,95]. The cooperative oncogenic effects of viral infections are considered plausible oncogenic drivers in different cancers [96]; coinfection by HPV and EBV stimulates EBV persistence either via latency, enhanced viral replication, or by triggering HPV oncogene expression [97]. Moreover, oncoproteins of HPV and EBV can lead to the development and progression of cancer via commonly linked signaling pathways including WNT/β-catenin, JAK/STAT/SRC, PI3k/Akt/mTOR, and/or RAS/MEK/ERK [34,96,98,99]. Interestingly, based on data reported by our lab and others, copresence of high-risk HPVs and EBV is involved in the onset and progression of various cancers including colorectal, head and neck, cervical, as well as breast cancer, by the initiation and/or amplification of the EMT event [42,44,45,46,47,93,98,100,101,102], thus indicating a similar role in the pathogenesis of human CRC malignancy. In addition, an in vitro study indicated that oncoproteins of HPV and EBV can cooperate to stimulate proliferation and invasion of breast cancer cells via Erk1/Erk2 and β-catenin pathways [102], suggesting a plausible mechanism for colorectal cancer cells.

## 4. Materials and Methods

### 4.1. Sample Collection and DNA Extraction

All samples were taken from formalin-fixed, paraffin-embedded (FFPE) tissues of surgically removed and pathologically confirmed colorectal carcinomas. Primary and naïve colorectal cancer samples from Lebanese patients were retrieved from the private pathology archive of Dr. Nawaf Jurdi, from Lebanon. All samples and histopathology reports were deidentified, and data were analyzed anonymously. In accordance with the ethical standards and the current Lebanese legislation, practicing physicians can orally inform all patients about the potential utilization of biopsy samples for research or secondary purposes. Therefore, written informed consent was not required. Additionally, the Institutional Biosafety Committee (IBC) approval was obtained from Qatar University (QU-IBC-2018/22) to investigate the presence of HPV and EBV in human CRCs.

All tumor samples were graded according to the American Pathologists Consensus Statement [48] using the two-tiered grading system (grade 1 and grade 2). Briefly, grade 1 (low-grade) combines well and moderately differentiated adenocarcinomas, while grade 2 (high-grade) refers to poorly differentiated and undifferentiated adenocarcinomas. The tumor stage is identified according to the American Joint Committee on Cancer (AJCC) TNM system (eighth edition) [49].

Punch samples of 2 mm thickness were obtained from the FFPE blocks of surgically removed and pathologically confirmed colorectal carcinomas. The Thermo Scientific GeneJET FFPE DNA Purification Kit was used to extract DNA from FFPE tissue samples in accordance with the manufacturer’s instructions (ThermoFisher Scientific, Waltham, MA, USA), as previously described by our group [47].

### 4.2. HPV and EBV Detection by PCR

Purified genomic DNA (25 ng) extracted from each sample was analyzed for HPV and EBV by PCR using specific primers for HPV types (16, 18, 31, 33, 35, 39, 45, 51, 52, 56, 58, 59, 66, and 68) of the E6/E7 region and for LMP1 gene of EBV, as previously described [47]. GAPDH was used as an internal control. Analysis was performed as previously described by our group [103,104].

The Invitrogen Platinum II Hot-Start Green PCR Master Mix (2×) (ThermoFisher Scientific, USA) was used to carry out PCR, as performed previously [47]. The PCR product from each exon was resolved using 1.5% agarose gel electrophoresis.

### 4.3. Tissue Microarray (TMA)

Tissue microarray was constructed as previously described by our group [42,44,100]. In brief, using a manual tissue arrayer (Beecher Instruments, Silver Spring, MD, USA), both control and cancer cases were implanted into a virgin paraffin TMA block.

A duplicate of 1 mm diameter TMA cores was sampled from areas of the tumor within donor blocks from a cohort of 107 samples (13 normal and 94 cancer samples). Subsequently, sections of 4 µm were sliced and subjected to hematoxylin and eosin staining on the initial slides to confirm the histopathologic diagnosis. Immunohistochemistry (IHC) assays were performed on the slides of the completed blocks against E6 and LMP1 of high-risk HPV and EBV, respectively.

### 4.4. Immunohistochemistry (IHC)

Immunohistochemical (IHC) analysis was performed on CRC samples by analyzing adjoining sections for E6 and LMP1 oncoproteins of high-risk HPV and EBV, respectively, using monoclonal antibodies for E6 of HPV (clones 1–4 and C1P5, Dako Agilent, Carpinteria, CA, USA and Calbiochem, ON, Canada) and LMP1 of EBV (clone CS1–4, Abcam, Abcam, Cambridge, MA, USA). Then, antigen retrieval in 10 mM citrate sodium citrate solution [pH 6] (Invitrogen, Carlsbad, CA, USA) and endogenous peroxidase activity was blocked with a solution of 3% hydrogen peroxide in methanol, and TMA slides were incubated for 35 min at 37 °C with primary antibody using a fully automated immunostainer (Ventana Medical System, Tuscon, AZ, USA). Prior to mounting, slides were counterstained with hematoxylin and eosin staining was completed in accordance with the manufacturer’s recommendations. Negative controls were processed by omitting the specific primary antibody for E6 and LMP1.

If cancer cells showed positivity in ≥1% of the cells, the tumors were considered positive for E6 and LMP1 [44]. LMP1 protein expression (EBV) was also analyzed in tumor-infiltrating lymphocytes and stromal cells [44]. However, since PCR is more sensitive, as compared to IHC, data from PCR were considered reliable and were used for correlation analysis.

### 4.5. Statistical Analysis

Statistical analysis was carried out using IBM Statistical Package for the Social Sciences (version 25). We also performed the χ^2^ test, along with Yates’ correction and Fisher’s exact tests, to assess the significance of HPVs and EBV correlation with clinicopathological data (gender, age, tumor location, stage, and grade). Graphs were plotted using GraphPad Prism software (version 8.4.3), and statistical significance was achieved at *p* < 0.05.

## 5. Conclusions

In conclusion, we explored in this study the presence/copresence of high-risk HPVs and EBV in CRC samples from Lebanon. We found that HPVs are dominant in CRC samples, while the presence of EBV was comparatively low. Furthermore, the copresence of both, HPVs and EBV, was observed in 28% of the cases; however, we did not find a correlation between copresence of HPVs and EBV with tumor grade or stage, which may be due to the limited number of samples in the cohort. Nevertheless, we believe that available HPV vaccines and upcoming EBV vaccines can be used to prevent part of CRC cases. Moreover, additional studies are vital to elucidate the underlying mechanisms of the cooperative role of high-risk HPVs and EBV in the initiation and/or progression of human CRC.

## Figures and Tables

**Table 1:** Clinicopathological characteristics of the cohort of patients with colorectal cancer.

Characteristic	Categories	Number (%)
Gender	Male	37 (34.6)
	Female	70 (65.4)
Age	≤50	32 (29.9)
	>50	75 (70.1)
Tumor Location	Recto-sigmoid colon	46 (49)
	Other parts of colon	48 (51)
Tumor Grade ^φ^	Grade 1 (low-grade)	72 (76.6)
	Grade 2 (high-grade)	22 (23.4)
Tumor Stage (pT) *	pT1	6 (6.4)
	pT2	5 (5.3)
	pT3	79 (84)
	pT4	4 (4.3)
Lymph Node Involvement (pN)	pN0	52 (55.3)
	pN1	20 (21.3)
	pN2	22 (23.4)
	pN3	0 (0)

^φ^ Tumor grade is set according to the College of American Pathologists Consensus Statement [48]. * Tumor stage is based on the American Joint Committee on Cancer (AJCC) TNM system (8th edition) [49].

**Table 2:** Correlation of EBV and HPV-subtypes in Lebanese colorectal cancer patients.

Samples	No. of Cases	High-Risk HPV Types								
		16	18	31	33	35	45	51	52	58
EBV (+)	27	15	24	7	4	17	9	11	11	20
EBV (−)	67	22	12	6	0	10	13	14	9	6
**Total**	94	37	36	13	4	27	22	25	20	26
***p*-value**		**0.07**	<0.0001 ***	**0.07**	0.006 **	<0.0001 ***	**0.24**	**0.09**	<0.0001 ***	<0.0001 ***

** indicates significant *p*-values (<0.01); *** indicates significant *p*-values (<0.001).
